# GRP78 protein metabolism in obese and diabetic rats: a study of its role in metabolic disorders

**DOI:** 10.1186/s13098-024-01255-6

**Published:** 2024-01-13

**Authors:** Kai Xi, Hua-Ping Li, Yue-Hui Wang, Yang-Yang Li, Lei Wang, Miao-Miao Zhang, Xi Zhang, Bing-Wen Xing

**Affiliations:** The First Affiliated Hospital, and College of Clinical Medicine of Henan University of Science and Technology, Luoyang, 471003 China

**Keywords:** Auditory brainstem response, Cochlea histology, Diabetic rat, Endoplasmic reticulum stress, Obese rat

## Abstract

**Objective:**

This study aimed to compare and analyze the expression and significance of the GRP78 protein in cochlear cell injury induced by a high glucose and high-fat diet in obese and diabetic rats.

**Methods:**

Male SD rats were randomly divided into two groups: normal (NC) and high-fat (HF) groups. The NC group was fed a standard diet for eight weeks, while the HF group received a high-glucose, high-fat diet. The HF group was further categorized into the obesity group (OB group) and the type II diabetes mellitus group (T2DM group). To induce a type II diabetes mellitus (T2DM) model, the T2DM group received an intraperitoneal injection of a small dose of STZ (45 mg/kg). After four weeks on the original diet, body weight, blood glucose, blood lipid levels, and auditory brainstem response (ABR) thresholds were measured. The cochlea was dissected, and its morphology was observed using HE staining. Immunohistochemistry and western blotting were utilized to examine the expression level of the GRP78 protein in the cochlea.

**Results:**

(1) The ABR threshold demonstrated a statistically significant difference between the T2DM group and the OB group (*P* < 0.05), as well as between the OB group and the NC group (*P* < 0.05). (2) Based on morphological comparisons from HE-stained sections, the T2DM group exhibited the most significant alterations in the number of cells in the spiral ganglion, the organ of Corti, and the stria vascularis of the cochlea. (3) The expression level of the GRP78 protein in the cochlea was higher in the T2DM group compared to the OB group (*P* < 0.05) and higher in the OB group compared to the NC group (*P* < 0.05).

**Conclusion:**

The findings indicate that the GRP78 protein plays a role in hearing loss caused by T2DM and hyperlipidemia. Moreover, T2DM is more likely than hyperlipidemia to be associated with hearing impairment.

## Introduction

 With improved living conditions and dietary changes, the incidence of metabolic diseases such as diabetes and obesity are on the rise [1]. Obesity can result in high cholesterol, high triglycerides, insulin resistance, increased peripheral vascular resistance, and so on, which can lead to hypertension, coronary heart disease, type II diabetes mellitus (T2DM), and an increase in the morbidity and mortality associated with these diseases [2–4]. Hyperlipidemia and diabetes have been shown to cause hearing loss, but the mechanism is still unclear [5, 6]. Long-term metabolic disorders such as hyperlipidemia and hyperglycemia can induce oxidative stress, activate multiple apoptotic signaling pathways, and cause cell damage [7]. Endoplasmic reticulum stresses (ERS) are currently being implicated in an increasing number of studies as a factor in hearing loss [8–10]. In the ERS process, the high expression of its marker protein 78 (glucose-regulated protein 78 (GRP78)), also known as immunoglobulin heavy chain binding protein (Bip), activates many caspase family factors, such as the high expression of caspase-12, resulting in apoptosis [11]. In this study, we investigated the expression pattern of the GRP78 protein within the cochlea. Our study delved into a comparative and analytical exploration of the ERS mechanism underlying hearing impairment in cases involving both obesity and diabetes.

## Materials and methods

### Experimental animals and feed

The study utilized 30 healthy, four-week-old male Sprague-Dawley (SD) rats weighing between 200 and 220 g, which were supplied by the Laboratory of the School of Basic Medical Science at Henan University of Science and Technology. The rats were provided unrestricted access to water and exposed to alternating cycles of natural day and night light, maintaining a controlled environment with temperatures ranging from 20 to 25 °C and relative humidity levels between 40% and 60%. Regarding their diet, they were fed a high-glucose and high-lipid feed composed of basic feed (69.5%), cholesterol (1%), sucrose (10%), and other ingredients (19.5%).

This study was conducted with approval from the Ethics Committee of The First Affiliated Hospital,and College of Clinical Medicine of Henan University of Science and Technology. All applicable international, national, and/or institutional guidelines for the care and use of animals were followed.

### Model establishment and grouping

After one week of adaptive feeding with basic feed, the rats were randomly divided into the normal control group (NC group, *n* = 10) and high-fat group (HF group, *n* = 20). The rats were fed basic feed, high glucose feed, and high fat feed for eight weeks each. When the average body weight of the HF group exceeded 20% of the average body weight of the NC group, it was deemed that the obesity model had been successfully established. The HF group was randomly divided into the obesity group (OB group, *n* = 10) and diabetes group (T2DM, *n* = 10). A low dose of STZ (45 mg/kg) was administered intraperitoneally to the T2DM group, while the OB group and the NC group received an equivalent intraperitoneal dose of citric acid buffer. Following four weeks of being on the original diet, blood samples were drawn from the tail vein to measure fasting glucose and lipid levels. The T2DM group was successfully modeled when the blood glucose level was > 11.1 mmol/L.

### Auditory brainstem evoked potentials

The rats were intraperitoneally injected with a 2% pentobarbital sodium solution at a dosage of 45 mg/kg in a designated soundproof room. Following successful anesthesia, the rats were positioned on an anatomical table. The recording electrode was carefully positioned under the skin at the middle cranial region of both ears, and the reference electrode was placed under the skin of the pinna of the ear being measured. The rats had an earphone inserted into their external auditory canal. The auditory brainstem response (ABR) threshold of the rats was measured using an evoked potential meter (Neuro-Audio Russia) [12]. The stimulation of tone pips was superimposed 1024 times, and the frequency range of the filtering was 100–3000 Hz. The frequency of stimulation was 21 times per minute. The stimulation intensity began at 90 dB SPL, decreased gradually by 10 dB SPL, and then decreased by 5 dB SPL as the level approached the ABR threshold. The ABR threshold was determined using the wave III threshold when the resistance was less than 3 kΩ.

### Preparation of cochlear specimens

Six cochleae were selected in each group. After inducing general anesthesia, the cochleae were carefully extracted from the sacrificed rats following decapitation. A thin needle was used to puncture open the round window membrane and the tip of the cochlea. Subsequently, four injections of 10% paraformaldehyde were administered into the cochlea’s tip using a latex dropper. Following immersion in the fixing solution overnight, the cochleae were rinsed with phosphate-buffered saline (PBS) and subjected to a two-week decalcification process using a 10% EDTA decalcification solution, with the solution being renewed every three days. After complete decalcification, the samples were routinely embedded in paraffin for subsequent tissue sectioning.

### HE staining [13]

Sections measuring 5 μm were sliced using a microtome, followed by a 10-minute dewaxing process with xylene I, II, and III, and subsequent hydration with gradient alcohol. Hematoxylin dye solution (obtained from Beijing Soleil Technology Co., Ltd.) was added drop by drop to the wet box, and the sections were allowed to react for four minutes. They were then rinsed thoroughly with running water until no residual blue color was visible. The cells were briefly treated with a differentiation solution containing 1% hydrochloric acid alcohol for 2 to 3 s, followed by a 30-minute rinse with running water to restore their blue color. Following the hematoxylin staining process, eosin dye solution (sourced from Beijing Soleil Technology Co., Ltd.) was added drop by drop for a duration of 3 min. The sections were then dehydrated and sealed for further analysis.

### Immunohistochemical experiment [14, 15]

After dewaxing the sections in water, 3% H2O2 was added dropwise to the sections in the dark at room temperature for 10 min, and the sections were then immersed and washed in PBS. The antigen retrieval process involved microwaving for 10 min, followed by incubation in a blocking solution comprising 5% goat serum for 30 min. After rinsing, the samples were coated with GRP78/Bip rabbit monoclonal antibody (BA2042-1, sourced from Beijing Soleil Technology Co., Ltd.) at a dilution ratio of 1:400 in PBS and left overnight at 4 °C. The following day, after rewarming, the samples were removed, rinsed, and then subjected to the dropwise addition of goat anti-rabbit IgG secondary antibody (from Beijing Soleil Technology Co., Ltd.) at a dilution ratio of 1:200 in PBS. Subsequently, the samples were placed in a 37 °C incubator for 30 min, followed by rinsing. Dropwise addition of DAB dye solution (sourced from Shanghai Epizyme Biological Medicine Co., Ltd.) was carried out under a microscope (Olympus BX41, Japan). Color development was monitored, and the reaction was terminated by rinsing the samples under running water once the color changed from colorless to yellow. Hematoxylin was then applied dropwise for counterstaining. After the color changed to blue, the sections were dehydrated and subsequently sealed for analysis.

### Western blot analysis of protein concentration

Four rats were chosen from each group. Following decapitation, the cochlea was extracted, rinsed with PBS, and immersed in liquid nitrogen for 5 min. Subsequently, the tissue was ground, and a protein lysis buffer was added. After ultrasonic homogenization, the protein was quantified using the BCA protein quantitative method. This was followed by incubation at 95 °C for 10 min and centrifugation at 12,000 revolutions per minute (r/min) for 15 min to obtain the protein extract. The protein was placed in the blocking solution after polyacrylamide gel electrophoresis and membrane transfer, and GRP78 antibody (1:1000) and internal reference–actin (1:5000) were added after shaker incubation, followed by overnight incubation at 4 °C. After being washed three times with Tris-buffered saline with Tween (TBST) buffer, the membrane was exposed to the secondary antibody for one hour. The relative expression level of the GRP78/Bip protein was calculated using ImageProPlus5 software (relative expression level of protein = gray value of the target protein/gray value of β-actin).

### Statistical analysis

SPSS25.0 software was used for statistical analysis. Quantitative data such as body weight, blood glucose level, and ABR threshold of rats in each group are expressed as mean ± standard deviation. One-way analysis of variance was used to compare multiple groups, and the LSD t-test was used to compare pairs of groups, with *P* < 0.05 indicating statistically significant differences.

## Results

### Changes in body weight and comparative analysis of blood glucose and lipid in rats

By the 8th week, the average body weights of the OB and T2DM groups had exceeded 20% of that in the NC group. By the 12th week, the blood glucose level in the T2DM group exceeded 11.1 mmol/L, indicating the successful establishment of the diabetic model. At this point, the body weight of the OB group was significantly higher than that of the NC group (*P* < 0.01), while the body weight of the T2DM group was notably lower than both the NC group and the OB group (*P* < 0.01). The T2DM group had significantly higher fasting blood glucose, total cholesterol (TC), and triglyceride (TG) values than the NC group and the OB group, and the OB group had higher values than the NC group (Table 1). A high-glucose and high-fat diet may cause metabolic disorders in the body over time.

**Table 1:** Body weight, blood glucose, and the lipid levels of rats in the three groups (*n* = 30)

Group	Weight (g)			Blood glucose level	Blood lipid level (mmol/L)	
	Week 1	Week 8	Week 12		Total cholesterol	Total triglyceride
NC group	228.1 ± 11.06	322.5 ± 12.59	413.9 ± 8.06	8.77 ± 1.44	1.63 ± 0.26	0.53 ± 0.22
OB group	229.1 ± 13.03	404.3 ± 8.31**	512.8 ± 12.92**	11.03 ± 1.67*	2.02 ± 0.47*	0.94 ± 0.55*
T2DM group	229.5 ± 12.21	404.6 ± 15.41**	298.9 ± 9.55**▫▫	19.93 ± 6.68**▫▫	2.59 ± 0.45**▫	1.74 ± 1.03**▫

*Indicates *P* < 0.05 as compared with the NC group;

**Indicates *P* < 0.01 as compared with the NC group

▫Indicates *P* < 0.05 as compared with that OB group

▫▫Indicates *P* < 0.01 as compared to OB group

### Comparison and analysis of ABR thresholds in rats

**Fig. 1:**
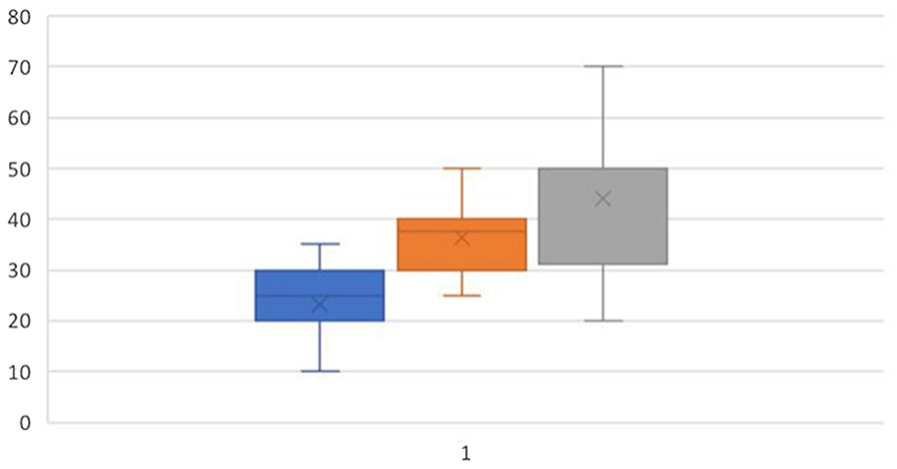
Comparison of ABR thresholds in the three groups of rats (*n* = 30). Number of cochleae = 20. ABR thresholds in OB group was significantly different (*P* < 0.05) from NC group. ABR thresholds in T2DM was significantly different (*P* < 0.05) from NC group, and OB group

Compared with the NC group, the ABR thresholds in the T2DM and OB groups were statistically significant (*P* < 0.05), while the ABR threshold in the T2DM group was statistically significant compared to the OB group. The ABR thresholds of the T2DM group were also significantly higher than those of the NC and OB groups, with the OB group exceeding the NC group (Fig. 1).

### Comparison and analysis of light microscope observation results

**Fig. 2:**
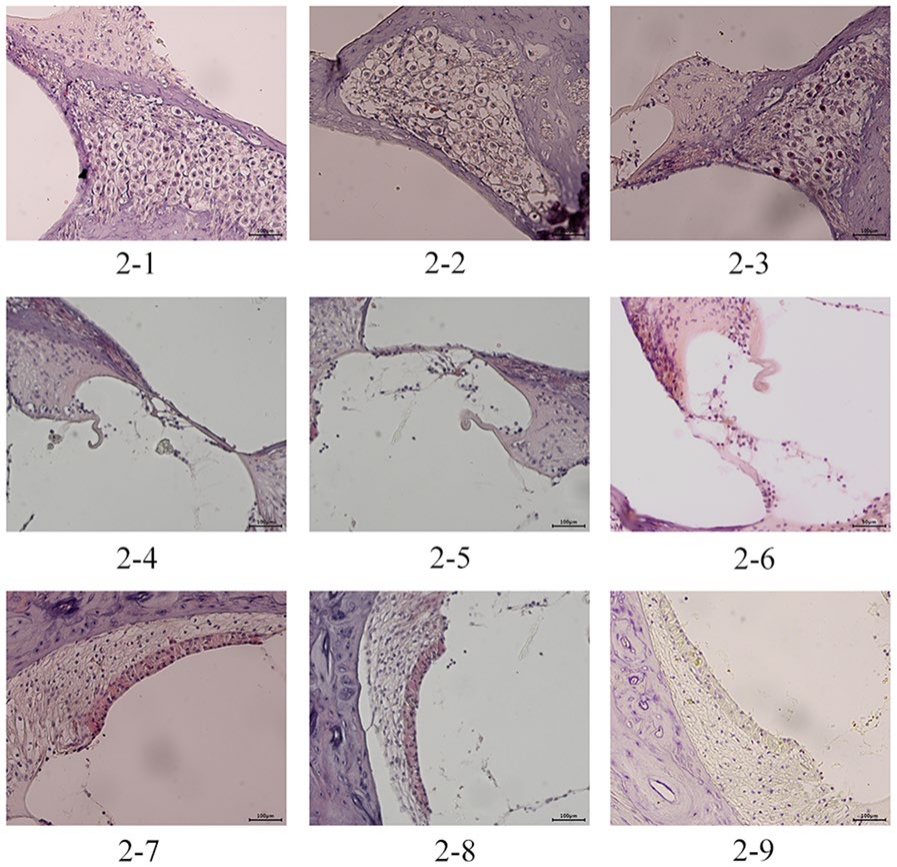
Morphological manifestations of the spiral ganglion, organ of Corti, and stria vascularis of the cochlea under a light microscope (×200).  1, 4, and 7 depict the spiral ganglion, organ of Corti, and stria vascularis in the NC group, respectively  2, 5, and 8 depict the spiral ganglion, organ of Corti, and stria vascularis in the OB group, respectively  3, 6, and 9 depict the spiral ganglion, organ of Corti, and stria vascularis in the T2DM group, respectively

1. Visually, the spiral ganglion cells in the OB group did not exhibit a significant reduction compared to the NC group. However, these cells appeared loosely arranged and disordered. Additionally, the outer hair cells in the organ of Corti displayed deformities and partial displacement. Observation of the striations revealed thicker capillary diameters, narrow lumens, and disorganized, apoptotic marginal cells (Fig. 2).

2. Compared to the NC group and the OB group, the spiral ganglion cells in the T2DM group were significantly diminished, and the cells were obviously disordered in arrangement, with the cytoplasm atrophying and the nucleus enlarging. Additionally, some hair cells outside of the organ of Corti underwent apoptosis, and the number of striated cells decreased significantly. The capillary lumen shrank, and the number of marginal cells decreased considerably (Fig. 2).

### Comparison and analysis of experimental results of immunohistochemistry

**Fig. 3:**
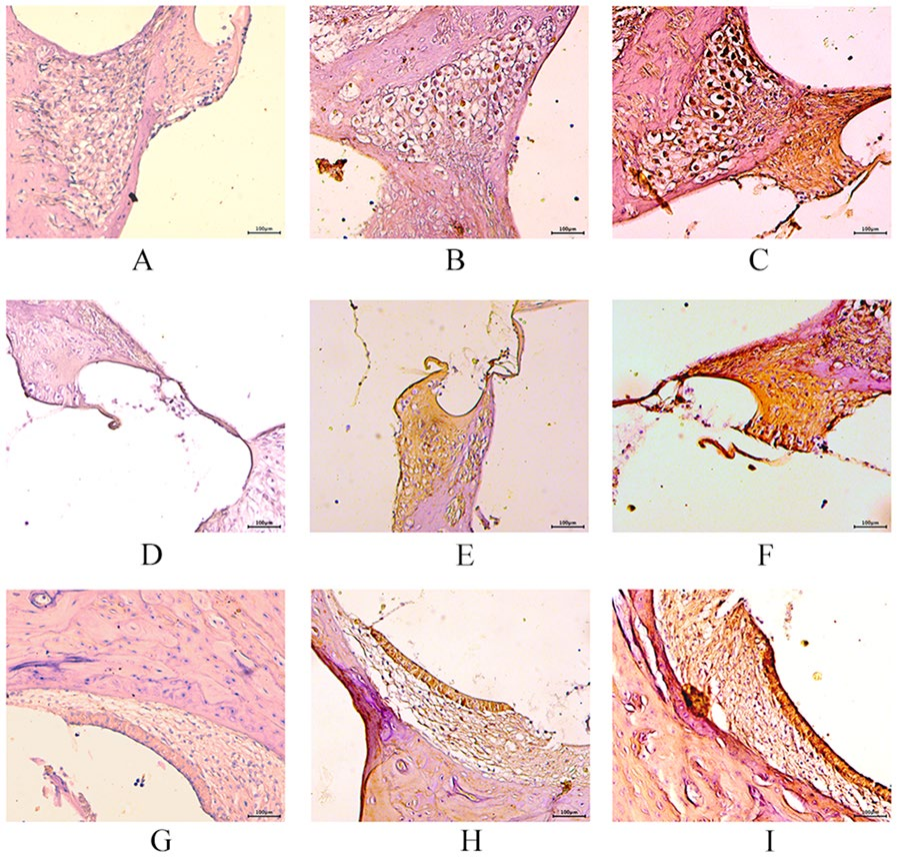
Immunohistochemical staining results of the spiral ganglion, organ of Corti, and stria vascularis of the cochlea under a light microscope (×200). ** A**, **D**, and **G** are the staining results of the spiral ganglion, organ of Corti, and stria vascularis in the NC group, respectively; the cytoplasm was faintly stained or not stained  **B**, **E**, and **H** are the staining results of the spiral ganglion, organ of Corti, and stria vascularis in the OB group, respectively; the cytoplasm was stained mildly or moderately  **C**, **F**, and **I** are the staining results of the spiral ganglion, organ of Corti, and stria vascularis in the T2DM group, respectively; the cytoplasmic staining is significantly deep and visible, and there are significantly more brownish-yellow particles distributed at ×200

In the NC group, the expression of the GRP78 protein was either weakly positive or negative in the spiral ganglion, organ of Corti, and stria vascularis. Conversely, in the OB and T2DM groups, the protein exhibited positive expression in the cytoplasm of the spiral ganglion. Positive expression results were more significant in the T2DM group than in the OB group (Fig. 3).

### Expression of GRP78 protein in rat cochlea

**Fig. 4:**
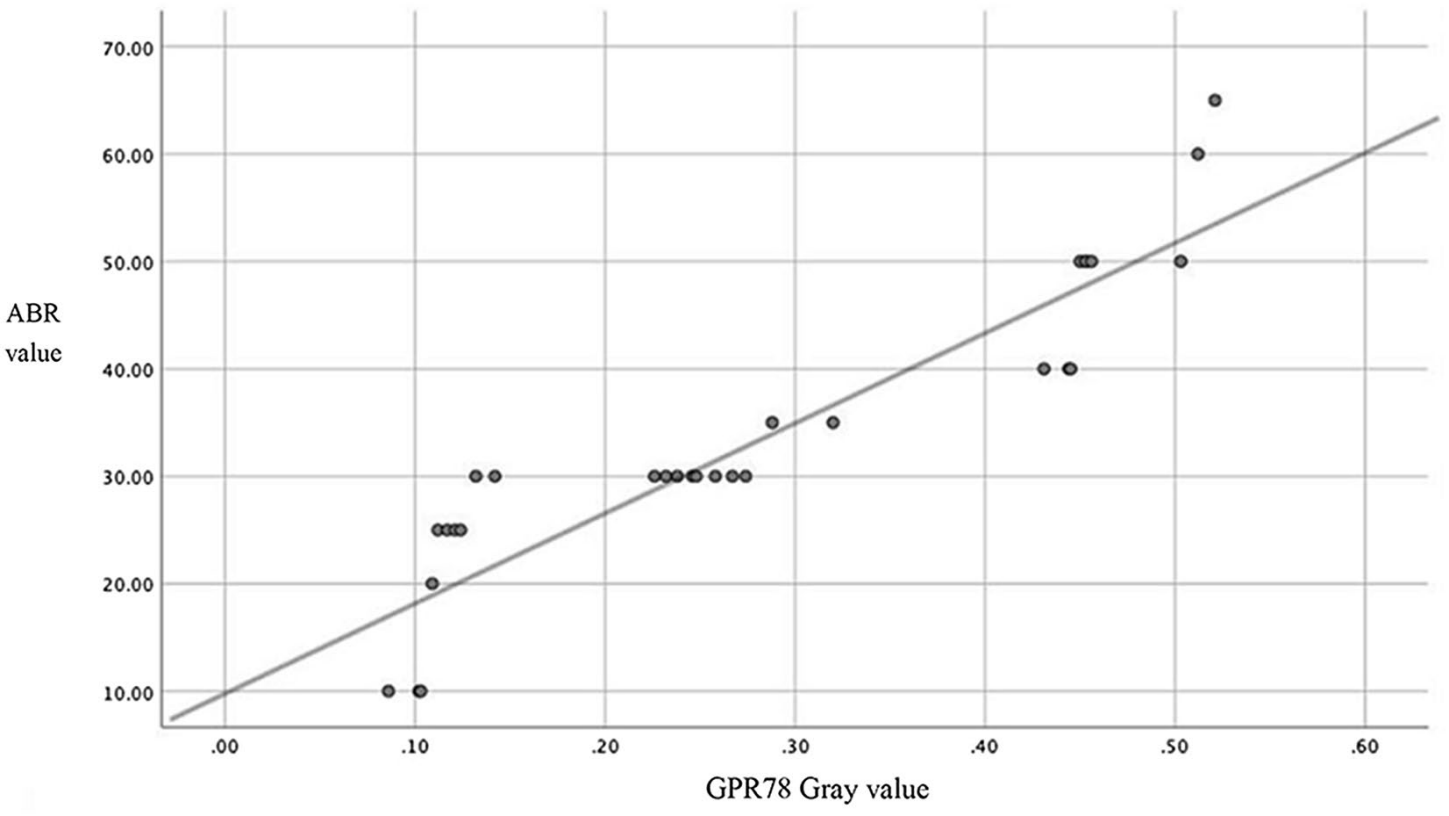
Correlation analysis between GRP78 protein and ABR threshold. Average immunohistochemical optical density (OD) values of rats in the three groups were analyzed. OD values in OB group was significantly different (*P* < 0.05) from NC group. OD values in T2DM was significantly different (*P* < 0.05) from NC group, and OB group

Image J software was used to analyze the optical density values of immunohistochemistry, including spiral ganglion, organ of Corti, and stria vascularis, of rats in each group. It was found that the darker the immunohistochemical staining, the stronger the absorbance. The optical density values of rats in the T2DM group were significantly higher than those in the OB group, and the values of the OB group were significantly higher than those of the NC group (*P* < 0.05) (Fig. 4).

### Expression of GRP78 protein in the cochlea detected by western blot

**Fig. 5:**
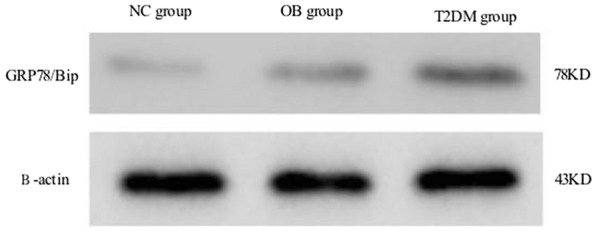
Western blot analysis of GRP78 and β-actin protein expression in the inner ear tissues of the three groups (*n* = 30)

**Fig. 6:**
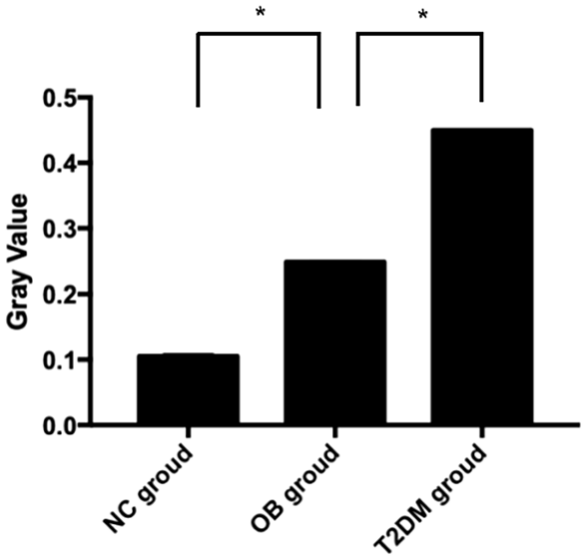
Comparison of GRP78 protein gray values in the three groups of rats (*n* = 30). *Indicates *P* < 0.05 as compared with the two groups

The GRP78 protein was expressed at a higher level in the T2DM group than in the OB group and the NC group, and more highly expressed in the OB group compared to the NC group. (Fig. 5) The expression of GRP78 in the OB group was significantly higher than that in the NC group (*P* < 0.05), and the expression of GRP78 in the T2DM group was significantly higher than that in the OB group (*P* < 0.05) (Fig. 6).

## Discussion

The GRP78 protein is an ERS chaperone. Its expression is significantly increased under ERS, so that it combines with misfolded and unfolded proteins in the endoplasmic reticulum to restore the correct conformation of proteins and enables proteins to continue to be correctly synthesized under cell stress, maintaining the homeostasis of endoplasmic reticulum calcium and the homeostasis [16]. Furthermore, GRP78 functions by facilitating the translocation of misfolded proteins to the endoplasmic reticulum, thereby ensuring the uninterrupted synthesis of proteins within cells experiencing stress conditions. GRP78 exists in the complex with Caspase7 and Caspase12 under normal conditions. Nevertheless, following ERS, GRP78 is dissociated from the complex and bound to the intraluminal unfolded protein, resulting in the activation and release of Caspase12 and apoptosis.

Obesity and diabetes have become a worldwide epidemic as a result of the modern lifestyle. However, studies indicate that hyperlipidemia and hyperglycemia resulting from long-term obesity are closely linked to hearing loss. In this study, we simulated the natural pathogenesis of human obesity and prepared obese and diabetic rat models using a high-fat and high-glucose diet, respectively combined with STZ in order to observe the expression of GRP78 protein, a molecular chaperone of ERS, in each group of cochleae, and investigated the molecular mechanism of hyperlipidemia and diabetes on inner ear damage.

The results of this study revealed that the ABR threshold of rats in the OB group was elevated. The organ of Corti, striations, and spiral ganglion exhibited more evident cell arrangement disorder, destruction, and apoptosis compared to rats in the NC group, and contained more GRP78 protein. Furthermore, numerous studies have confirmed that dyslipidemia is a risk factor for hearing loss [17–21]. Fuji et al. developed animal models of ERS-induced cochlear cell injury and discovered that the ERS chaperone in in cochlear cells was significantly elevated, indicating that ERS may be responsible for hearing loss [22]. The results of this study are comparable to those of previous studies. However, additional animal experiments in this study confirmed the changes in the microstructures of inner ears with hearing loss caused by dyslipidemia and its correlation with ERS. We hypothesize that the elevated levels of blood lipids and altered hemorheology in obese rats led to increased blood viscosity and flow velocity. Consequently, this condition resulted in tissue hypoxia, triggering ERS in the spiral ganglion, organ of Corti, and striated vascular cells. Additionally, it led to the release of apoptosis-inducing factors, causing cell atrophy, degeneration, and eventual apoptosis, culminating in hearing loss. Rats in the T2DM group exhibited higher ABR thresholds compared to those in the NC and OB groups. Upon the administration of a low dose of STZ, rats in the T2DM group demonstrated significantly elevated levels of blood glucose, serum TG, and serum TC in comparison to the OB group. This observation suggests that upon transitioning to a diabetic state, obese rats experienced more severe dysfunction in lipid metabolism. In addition, the cells in the organ of Corti, striations, and spiral ganglion demonstrated more prominent cell destruction and apoptosis than those in the OB group. Strong expression of the GRP78 protein was found in the cytoplasm of the organ of Corti, striations, and spiral ganglion. In their research, Jia et al. discovered ERS in the cochlear hair cells of mice with type II diabetes [10]. Consistent with the experimental findings, ERS may increase the defect rate of cochlear outer hair cells and the hearing threshold of diabetic mice. Long-term hyperlipidemia, glucosamine pathway induced by diabetic hyperglycemia, and ERS induced by insulin resistance of macrophages are hypothesized to result from diabetes. This leads to many GRP78 proteins to separate from the complex and combine with the unfolded proteins in the cavity, resulting in the activation and release of Caspase12, as well as the disordered arrangement, destruction, and apoptosis of the organ of Corti, striations, and spiral ganglion cells in the cochlea, which ultimately leads to hearing loss. Liu et al. established the in vitro ERS model using hair cells treated with tunicamycin [23]. The observation that alleviating ERS can mitigate sensorineural hearing loss suggests a clear association between ERS and hearing impairment. Research conducted by Oishi et al. revealed that ERS, when mitigated by XBP1, could potentially mask aminoglycoside neurotoxicity at the organismal level, including hearing loss [24]. Increased expression of the ERS protein molecular chaperone and hearing loss have been shown to have a direct or indirect relationship, based on western blot and reverse transcription PCR analysis.

This study has several limitations: (1) The limited number of experimental animals diminishes the persuasiveness of the results. (2) Due to experimental constraints, dynamic observation and comparison were not conducted, restricting the comprehensive understanding of the phenomena. (3) Detailed characterization of cochlear hair cells, particularly the basement membrane, was hindered due to equipment and resource limitations. (4) To strengthen the quantitative data, a more robust study design, such as incorporating stereological studies, is necessary for further confirmation and validation of the findings.

## Conclusion

Obesity and T2DM can induce ERS in cells, leading to cell apoptosis, morphological and structural changes in the cochlea, and varying degrees of hearing loss.

The coexistence of hyperglycemia and hyperlipidemia results in more profound damage to cochlear cells. This discovery establishes a crucial experimental foundation for the early intervention of hearing loss stemming from diabetes and hyperlipidemia. Furthermore, it signifies the involvement of the GRP78 protein, a molecular component associated with ERS, in the mechanisms underlying hyperlipidemia-induced hearing impairment in diabetes. Consequently, these findings offer a molecular framework for the treatment of diabetes-related hearing loss. Owing to constraints related to laboratory conditions and time, we regrettably could not administer the intervention treatment to the obese and diabetic rats. Additionally, we were unable to investigate the extent of hearing recovery post-treatment and the expression of GRP78/Bip proteins in their cochlea. This limitation prevented us from providing more direct evidence in our study.

## Data Availability

All data generated or analyzed during this study are included in this article. Further enquiries can be directed to the corresponding author.
